# Extravascular Cyanoacrylate “Embolization” for the Treatment of Vesico-Vaginal Fistula: A Minimally Invasive Alternative

**DOI:** 10.3390/diagnostics16203377

**Published:** 2026-10-17

**Authors:** Antonio Vizzuso, Maria Vittoria Bazzocchi, Antonio Spina, Pasquale Maria Berrino, Roberta Gunelli, Emanuela Giampalma, Matteo Renzulli

**Affiliations:** 1 Radiology Unit, Morgagni-Pierantoni Hospital, AUSL Romagna, 47121 Forlì, Italy; 2 Urology Unit, Morgagni-Pierantoni Hospital, AUSL Romagna, 47121 Forlì, Italy; 3 Department of Medical and Surgical Sciences, University of Bologna, 40100 Bologna, Italy

**Keywords:** cyanoacrylate, N-butyl cyanoacrylate, vesico-vaginal fistula, urinary fistola, catheter-direct therapy, minimally invasive treatment, voiding cystourethrography, extravascular embolization

## Abstract

Vesico-vaginal fistula (VVF) is a debilitating complication commonly occurring after gynecologic surgery and frequently associated with significant morbidity and impaired quality of life. Although surgical repair remains the standard treatment, management strategies remain heterogeneous, while conservative approaches often require prolonged urinary diversion with variable success rates. We report a case of persistent post-surgical VVF successfully treated with image-guided embolization using N-butyl cyanoacrylate methylsulfone (NBCA; Glubran 2, GEM; Viareggio, Italy) through a transvesical catheter-based approach after failure of conservative management. This case illustrates the potential role of interventional radiology in the management of selected VVFs, suggesting that image-guided fistula embolization may represent a minimally invasive treatment option when conservative management fails and surgical reintervention is undesirable or can be deferred.

**Figure 1:**
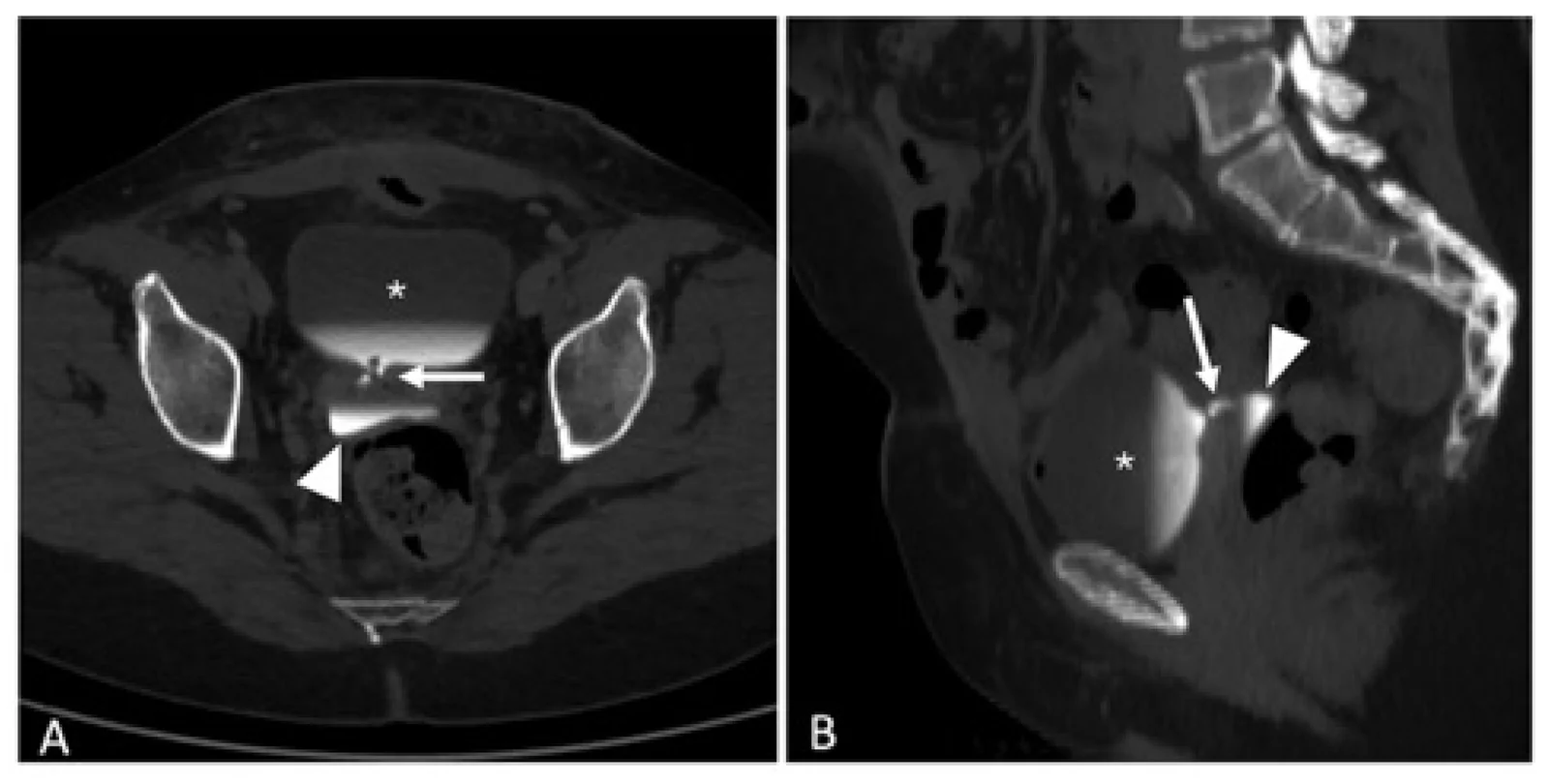
A 50-year-old woman with obesity (body mass index of 32 kg/m^2^), smoker, with a history of previous cesarean delivery, underwent total hysterectomy with bilateral adnexectomy for symptomatic uterine leiomyomas causing menorrhagia. The patient was perimenopausal with infrequent menstrual cycles and had declined hormonal treatment with progestins; considering her age and perimenopausal status, bilateral adnexectomy was performed. Surgery was technically challenging because of multiple large leiomyomas and dense adhesions between the bladder and the uterus related to the previous cesarean section, which made identification of the anatomical planes more difficult. However, no intraoperative bladder injury was recognized. Approximately 10 days after surgery, the patient developed continuous watery vaginal discharge. Speculum examination showed an apparently intact vaginal vault without visible defects. A methylene blue test performed by instillation into the bladder through a Foley catheter, with gauze placed in the vagina, showed no evidence of leakage. Transabdominal and transvaginal ultrasound also demonstrated an apparently intact vaginal vault. Because of persistent symptoms despite the negative initial examinations, computed tomography (CT) urography was performed; axial (**A**) and sagittal (**B**) images in urographic phase demonstrate opacification of the bladder (asterisks) with contrast leakage into the vaginal vault (arrowhead) through an approximately 18-mm-long fistulous tract (arrow) with an oblique course. The right ureteral orifice was located approximately 9 mm lateral to the fistulous opening. Initial management consisted of planned bilateral urinary diversion with percutaneous nephrostomy placement and ureteral stenting to promote fistula healing. Following successful placement of the right percutaneous nephrostomy and ureteral stent, the patient declined placement of the contralateral nephrostomy, both at the time of the procedure and during subsequent days; consequently, urinary diversion was achieved unilaterally.

**Figure 2:**
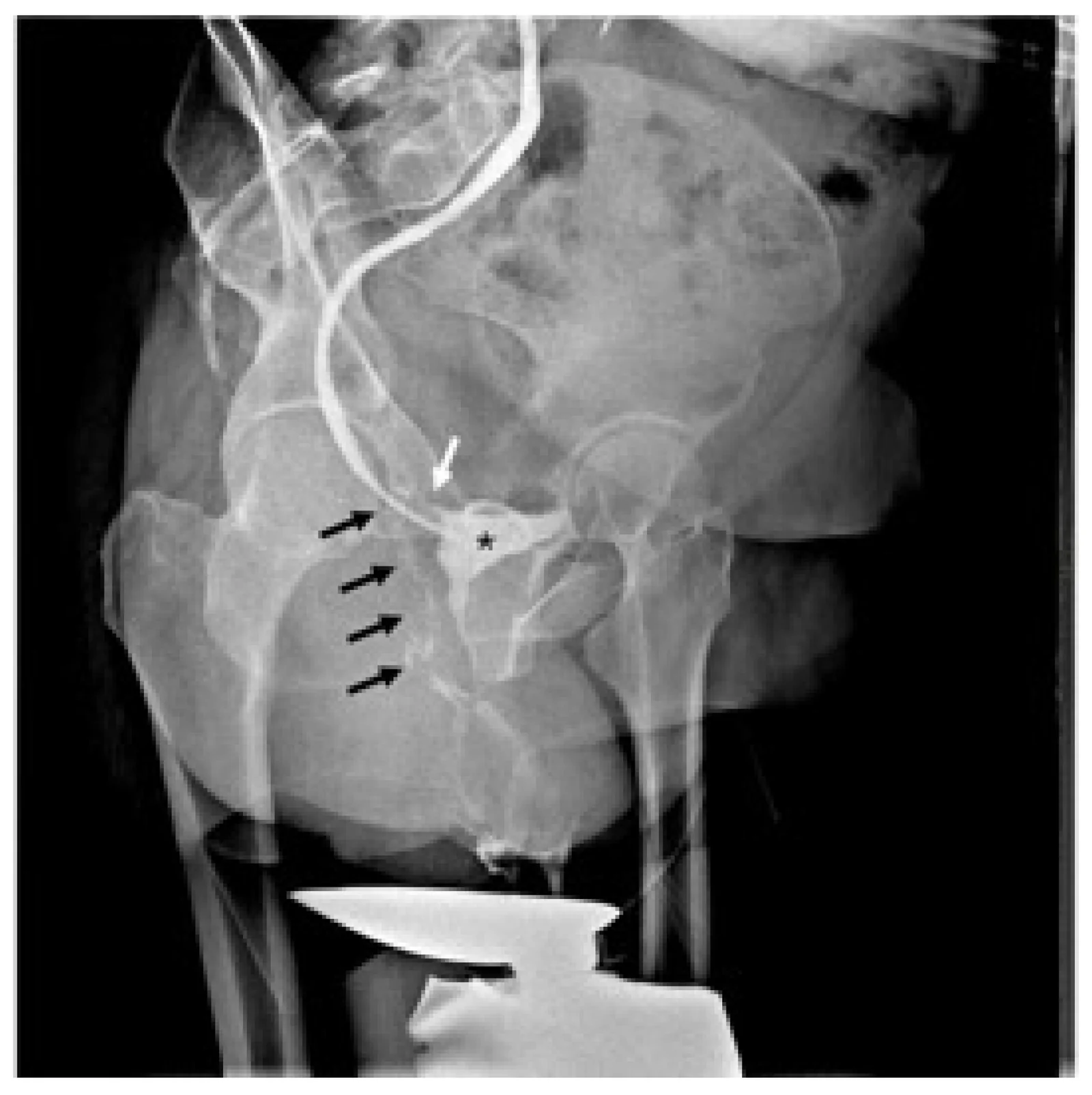
Voiding cystourethrography (VCUG) image in parasagittal projection obtained during the voiding phase demonstrates persistent, though small, passage of contrast medium from the bladder (black asterisks) into the vaginal cavity (black arrows) through a fistulous tract (white arrow) arising from the posterior bladder wall. Given the persistence of urinary leakage after two months despite urinary diversion, the patient was evaluated by a urogynecologist. Further surgical repair was discussed as a treatment option; however, considering the patient’s significant discomfort and reluctance to undergo an additional surgical procedure, a minimally invasive treatment was proposed. After multidisciplinary discussion, a fluoroscopy-guided catheter-based approach was selected based on local expertise.

**Figure 3:**
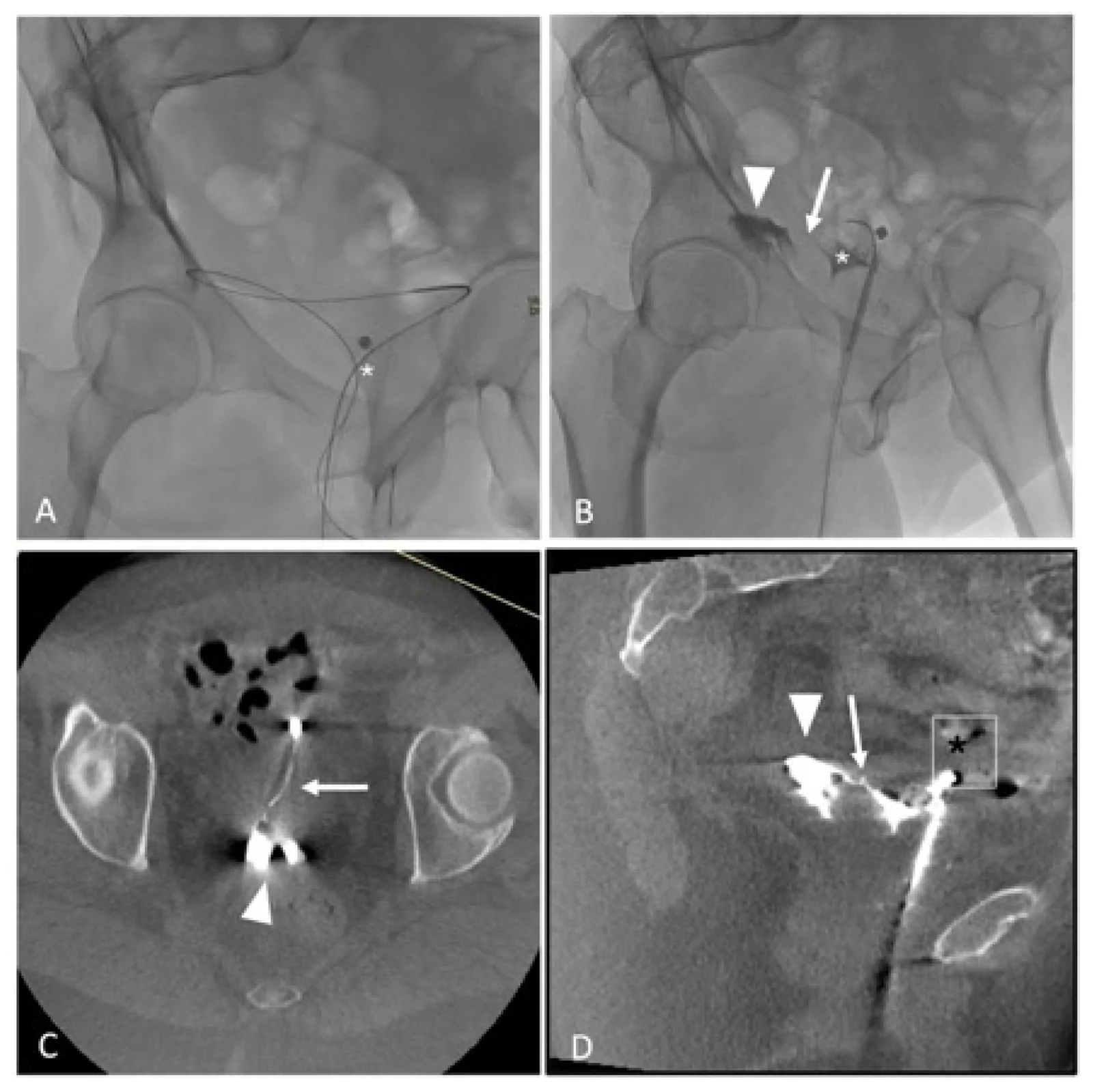
(**A**) Fluoroscopic image obtained during the procedure demonstrating the use of a 16-Fr Foley catheter as transurethral access to the bladder. A 0.035-inch guidewire and a 4-F diagnostic catheter are advanced through a side hole adjacent to the balloon (white asterisks). Once the bladder lumen was reached using this minimally invasive approach, after multiple attempts, the guidewire was subsequently navigated across the VVF tract and externalized through the vaginal opening, confirming successful fistula traversal tract. Then, the 4F catheter was advanced into the vaginal dome. (**B**) Following selective catheterization of the fistulous tract (arrow), a 2.7-Fr microcatheter was advanced to allow controlled delivery of the embolic agent. The microcatheter was flushed with 5% dextrose solution before embolization. NBCA mixed with Lipiodol (Guerbet, Villepinte, France) at a 1:2 ratio was subsequently administered under continuous fluoroscopic monitoring using a slow, continuous injection. The mixture was injected until adequate fluoroscopic filling of the fistulous tract (arrow) was achieved, including the vaginal dome (arrowhead), and the vesical orifice of the fistula (white asterisks). Post-procedural cone-beam CT images in the axial (**C**) and sagittal (**D**) planes confirm successful embolization, demonstrating hyperdense Lipiodol cast material along the fistulous tract (arrow), extending into the vaginal vault (arrowhead) and adjacent bladder (black asterisks). The bladder catheter was left in place for three days to ensure continuous bladder drainage during the early post-treatment period.

**Figure 4:**
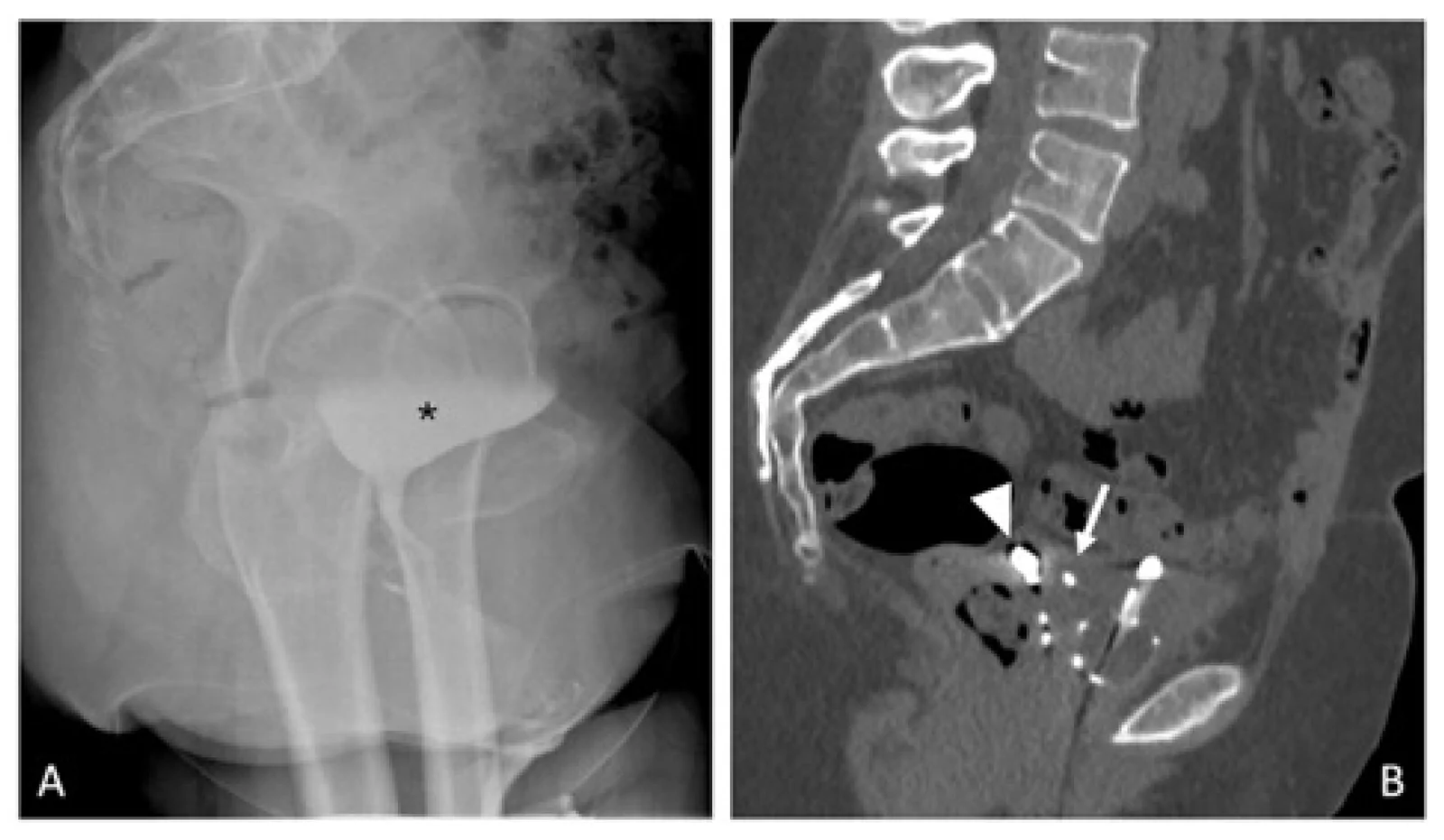
In the absence of fistula-related symptoms, clinical and (**A**) VCUG follow-up demonstrated normal bladder emptying (black asterisks) without evidence of contrast leakage into the vaginal cavity, confirming resolution of the VVF. No standardized dye test, pad test, or voided-volume measurement was performed. (**B**) Early post-procedural unenhanced CT scan showed residual hyperdense Lipiodol deposits along the treated fistulous tract (arrow) and within the vaginal vault (arrowhead), as well as small intravesical deposits surrounding the Foley catheter balloon. At six-month follow-up, the patient remained asymptomatic, and unenhanced CT demonstrated complete disappearance of the previously visible radiopaque deposits. VVF is a debilitating condition characterized by an abnormal communication between the bladder and the vagina, resulting in continuous urinary leakage and substantial impairment of quality of life. Most VVFs are secondary to gynecologic surgery, with approximately 0.8 per 1000 hysterectomies [1]. Diagnosis is generally based on clinical assessment and direct visualization of the fistulous tract, complemented by cystoscopy and contrast-enhanced urinary tract imaging to define fistula location and anatomy [1]. Although surgical repair remains the cornerstone of treatment achieving high success rates in experienced hands, optimal timing and approach remain controversial, and no universally accepted, evidence-based guidelines currently define a standardized management algorithm [2]. Multiple different surgical routes like Latzko repair, open transabdominal, transvaginal, laparoscopic, robotic, transurethral endoscopic with or without tissue interposition have been described [3]. Endoscopic approaches using direct injection of tissue adhesives into the fistulous tract, including fibrin glue and cyanoacrylate, have also been reported [4,5]. Conservative management with continuous urinary drainage has been proposed, typically including percutaneous nephrostomy, indwelling bladder catheterization, and ureteral stenting, especially in early-detected and small fistulas [3]. Bladder drainage alone has been described as a potential first-line conservative strategy, particularly when instituted early after the causative insult. However, spontaneous closure rates reported in the literature are low (7–15%) and vary across studies due to heterogeneity in fistula characteristics and treatment protocols, and healing may require prolonged catheterization [6]. During this period, persistent urinary leakage may significantly impair the patient’s physical comfort and psychosocial well-being, rendering prolonged expectant management clinically and emotionally burdensome. In this clinical setting, interventional radiology may offer minimally invasive alternatives. Liquid embolic agents, including cyanoacrylate-based glues and non-adhesive agents such as ethylene–vinyl alcohol copolymer, have emerged as promising options for fistula occlusion, providing rapid and potentially definitive tract sealing, as reported in recent experiences with urinary fistulas [2,3,7]. In our clinical practice, this approach is being explored in cases in which urinary diversion alone results in prolonged fistula persistence and continued symptoms, with the aim of achieving earlier interruption of urinary leakage and, consequently, more rapid symptom relief while preserving physiological urinary continuity. Compared with previously reported endoscopic injection techniques [4,5], this approach allowed guidewire-assisted traversal and selective microcatheterization of the fistulous tract, with controlled delivery and real-time fluoroscopic monitoring of the NBCA–Lipiodol mixture. Previous reports have described transrenal ureteral occlusion using vascular plugs in patients with lower urinary tract fistulas, particularly in complex or palliative contexts. However, these approaches are primarily designed to divert urine rather than restore physiological urinary flow by directly sealing the fistulous tract [8]. More recently, extravascular applications of embolic agents have expanded the scope of minimally invasive management in the genitourinary tract. Extravascular use of cyanoacrylate has been reported in different clinical settings, including pancreatic, esophageal, and urinary fistulas, supporting its role as a sealing agent [9]. Upon contact with biological fluids, cyanoacrylate undergoes rapid polymerization, resulting in solidification of the adhesive and mechanical occlusion of the fistulous tract. In this context, NBCA embolization may potentially be considered as a local treatment strategy for selected small residual fistulas, with the aim of interrupting persistent urinary leakage and promoting tract closure. Several technical features of this approach can be considered based on the present case. The procedure allowed direct treatment of the fistulous tract using transurethral access through the bladder catheter, without the need for an additional percutaneous access. Moreover, it provided early occlusion of the fistulous tract while preserving physiological urinary continuity. At six-month follow-up, unenhanced CT demonstrated complete radiological disappearance of the previously visible radiopaque deposits. However, these potential advantages should be interpreted cautiously, as they are derived from a single clinical experience and require confirmation in larger patient series. Potential limitations and complications of this technique should also be considered. First, the adhesive properties of cyanoacrylate and the associated local tissue reaction may theoretically make subsequent surgical dissection more challenging if surgical repair becomes necessary after treatment failure. The limited and selective deposition of the embolic agent within the fistulous tract was intended to minimize involvement of the surrounding tissues, although the potential impact of cyanoacrylate-related tissue reaction on subsequent surgical repair remains unknown. Second, small fragments of the NBCA–Lipiodol cast could potentially detach and migrate into the bladder lumen, where they might act as a nidus for stone formation and cause lithiasis-related symptoms. Maintaining the Foley catheter during the early post-procedural period may facilitate drainage of small intravesical fragments. In our patient, no lithiasis-related or other procedure-related symptoms were observed during follow-up, and the small intravesical radiopaque deposits observed immediately after the procedure were no longer detectable on unenhanced CT at six months. Nevertheless, these potential complications should be considered when selecting patients and planning the procedure, and their actual incidence cannot be determined from a single case. In the absence of clear, evidence-based guidelines for VVF management, treatment must be individualized. However, given the single-case nature of this report and the limited follow-up, no conclusions regarding long-term durability, comparative efficacy, or generalizability can be drawn. Larger studies with longer follow-up are needed to determine the safety, efficacy, durability, and appropriate patient selection criteria for this technique. However, this case illustrates a novel fluoroscopy-guided, microcatheter-based approach for selective NBCA delivery into a vesicovaginal fistulous tract, suggesting a potential minimally invasive treatment option in selected patients, when conservative measures fail and surgical intervention is undesirable or can be reasonably deferred.

## Data Availability

Data are contained within the article.

## Abbreviations

The following abbreviations are used in this manuscript:

VVF	Vesico-vaginal fistula
NBCA	N-butyl cyanoacrylate
CT	Computed Tomography
